# Beverage Intake Assessment Questionnaire: Relative Validity and Repeatability in a Spanish Population with Metabolic Syndrome from the PREDIMED-PLUS Study

**DOI:** 10.3390/nu8080475

**Published:** 2016-07-30

**Authors:** Cíntia Ferreira-Pêgo, Mariela Nissensohn, Stavros A. Kavouras, Nancy Babio, Lluís Serra-Majem, Adys Martín Águila, Andy Mauromoustakos, Jacqueline Álvarez Pérez, Jordi Salas-Salvadó

**Affiliations:** 1 Human Nutrition Unit, Biochemistry Biotechnology Department, Faculty of Medicine and Health Sciences, Universitat Rovira i Virgili, IISPV (Institut d’Investigació Sanitària Pere Virgili); Hospital Universitari de Sant Joan de Reus, C/Sant Llorenç, 21, Reus 43201, Spain; cintia.ferreira@iispv.cat (C.F.-P.); nancy.babio@urv.cat (N.B.); 2 CIBEROBN (Centro de Investigación Biomédica en Red Fisiopatología de la Obesidad y Nutrición), Instituto de Salud Carlos III, Av. Monforte de Lemos, 3-5. Pabellón 11. Planta 0, Madrid 28029, Spain; mnissensohn@acciones.ulpgc.es (M.N.); lluis.serra@ulpgc.es (L.S.-M.); jalvarez@proyinves.ulpgc.es (J.Á.P.); 3 Research Institute of Biomedical and Health Sciences, University of Las Palmas de Gran Canaria, Paseo Blas Cabrera Felipe “Físico” (s/n), Las Palmas de Gran Canaria 35016, Spain; 4 Hydration Science Lab, 155 Stadium dr-HPER 308Q, University of Arkansas, Fayetteville, AR 72701, USA; kavouras@uark.edu; 5 Clinical Analysis Unit, University Hospital of Gran Canaria Dr. Negrin, Barranco de la Ballena, s/n, Las Palmas de Gran Canaria 35010, Spain; maradys47@gmail.com; 6 Agricultural Statistics Lab, 101 Agricultural Annex Building, University of Arkansas, Fayetteville, AR 72701, USA; amauro@uark.edu

**Keywords:** relative validity, repeatability, fluid questionnaire, beverage, PREDIMED-PLUS study, Spain

## Abstract

We assess the repeatability and relative validity of a Spanish beverage intake questionnaire for assessing water intake from beverages. The present analysis was performed within the framework of the PREDIMED-PLUS trial. The study participants were adults (aged 55–75) with a BMI ≥27 and <40 kg/m^2^, and at least three components of Metabolic Syndrome (MetS). A trained dietitian completed the questionnaire. Participants provided 24-h urine samples, and the volume and urine osmolality were recorded. The repeatability of the baseline measurement at 6 and 1 year was examined by paired Student’s *t*-test comparisons. A total of 160 participants were included in the analysis. The Bland–Altman analysis showed relatively good agreement between total daily fluid intake assessed using the fluid-specific questionnaire, and urine osmolality and 24-h volume with parameter estimates of −0.65 and 0.22, respectively (*R*^2^ = 0.20; *p* < 0.001). In the repeatability test, no significant differences were found between neither type of beverage nor total daily fluid intake at 6 months and 1-year assessment, compared to baseline. The proposed fluid-specific assessment questionnaire designed to assess the consumption of water and other beverages in Spanish adult individuals was found to be relatively valid with good repeatability.

## 1. Introduction

Nowadays, estimating the total fluid intake and real beverage pattern of a population may be considered as a real challenge in nutritional epidemiology. The associations between hydration, water, or beverage intake with health or disease has recently become an important area of research [1,2]. Several authors have assessed the relationship between the consumption of beverages and specific outcomes: for example, the intake of sugar-sweetened beverages (SSBs) and metabolic syndrome (MetS) or type 2 diabetes (T2DM) [3], hypertension [4,5,6] and other cardiometabolic variables [7]; or the intake of drinking water and its relationship to cardiovascular diseases (CVD) [8]. However, the results in some cases are controversial [9,10,11] and it is probably partially attributable to the difficulties in assessing the real fluid pattern [12]. Water is an essential nutrient for life [13] and the research on its contribution to human health is very important, so it is essential that the technique used to assess the consumption of different types of beverage is sufficiently sensitive. 

To evaluate total fluid intake (all drinking water and beverages), it is common to use food frequency questionnaires (FFQ) or 24-h recall [14,15]. However, these questionnaires were mainly designed to evaluate food intake, and not fluid consumption as a whole. In addition, most food records or dietary recalls do not evaluate the consumption of drinking water because they do not provide calories. The assessment of beverage intake in recent years has mostly focused on SSBs and alcoholic drinks [16,17]. For this reason, and also because fluids are often consumed between meals and are not perceived as a food, fluid intake tends to be underestimated by the individual and the interviewer [3,18,19,20].

In 2010, Hedrick and coworkers published a questionnaire designed to assess the consumption of different types of beverage in the American population [21]. However, to the best of our knowledge, there is no standardized and validated questionnaire in Spanish that has been developed as a research tool for the specific assessment of beverage intake. 

For this reason, the main aim of the present study was to assess the repeatability and the relative validity of a new fluid-specific questionnaire designed to measure the habitual consumption of drinking water and different types of beverages in a Spanish population.

## 2. Material and Methods

### 2.1. Subjects and Design

The present analysis was performed within the framework of the PREDIMED-PLUS trial, the design of which has been described elsewhere [22]. Briefly, the PREDIMED-PLUS is a large, multicenter, parallel group, randomized and controlled clinical trial designed for evaluating the safety and effectiveness of a multifaceted intervention program for alleviating excessive cardiovascular morbidity and mortality in overweight and obese individuals. 

The primary endpoint of the PREDIMED-PLUS trial is to determine the effect on CVD morbidity and mortality of an intensive weight loss intervention program based on an energy-restricted traditional Mediterranean diet (MedDiet), increased physical activity and behavioral therapy in comparison with an intervention based on traditional Mediterranean diet advice (energy-unrestricted MedDiet) and traditional health care for CVD prevention.

All participants provided written informed consent, and the PREDIMED-PLUS protocol and procedures were approved by the Institutional Review Board Comité de Ética de Investigación Clínica del Hospital Universitario de Gran Canaria Dr. Negrín (code 130093, 30 January 2014) and Comité Ètic d’Investigació Clínica del Hospital S. Joan de Reus (code 13-07-25/7proj2, 25 July 2013). The trial is registered at clinicaltrials.gov; identifier: ISRCTN89898870.

The study participants were adult men aged 55–75 and women aged 60–75 with a body mass index (BMI) ≥27 and <40 kg/m^2^ and who met at least three of the following criteria for the MetS: abdominal obesity for European individuals (waist circumferences ≥88 cm in women and ≥102 cm in men), hypertriglyceridemia (≥150 g/dL) or drug treatment for high plasma triglyceride (TG) concentration, low high-density lipoprotein (HDL)-cholesterol (<50 mg/dL in women and <40 mg/dL in men), high blood pressure (systolic blood pressure ≥130 mmHg or diastolic blood pressure ≥85 mmHg) or antihypertensive drug treatment, or high fasting glucose (≥100 mg/dL) or drug treatment for T2DM. MetS was defined in accordance with the updated harmonized criteria of the International Diabetes Federation and the American Heart Association and National Heart, Lung and Blood Institute [23]. 

The analysis included a total random sample of 160 individuals randomized to the PREDIMED-PLUS trial from the Reus and Las Palmas de Gran Canaria centers. 

### 2.2. Assessment of Fluid Intake

**Figure 1:**
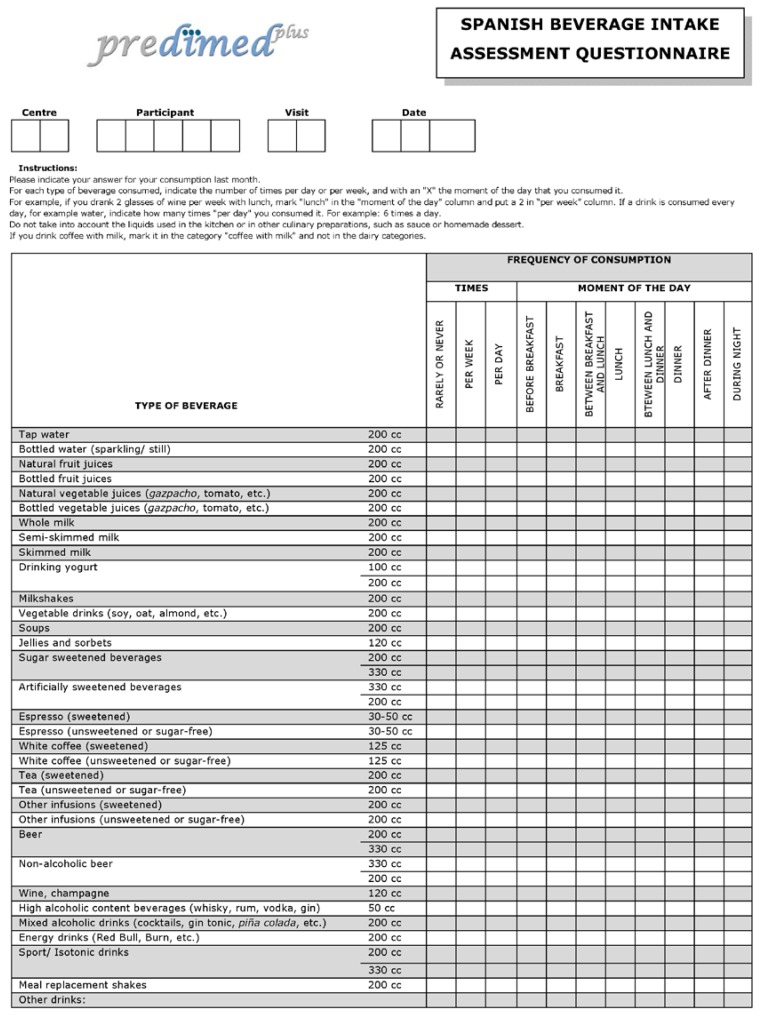
The beverage intake assessment questionnaire in English (translated version and not validated tool).

A trained dietician, on behalf of participants at an interview, filled in the fluid-specific questionnaire, recording the daily and weekly consumption of different types of beverage over the previous month (Figure 1 in English and Supplementary materials Figure S1 in Spanish). The average daily fluid intake from beverages was estimated on the basis of servings of each type of beverage. The questionnaire items on beverages included: tap water, bottled water, natural fruit juices, bottled fruit juices, natural vegetable juices, bottled vegetable juices, whole milk, semi-skimmed milk, skimmed milk, drinking yogurt (100 and 200 cc), milkshakes, vegetable drinks, soups, jellies and sorbets, sugar-sweetened beverages (SSBs) (200 and 330 cc), artificially-sweetened beverages (ASBs) (200 and 330 cc), espresso (sweetened and unsweetened), white coffee (sweetened and unsweetened), tea (sweetened and unsweetened), other infusions (sweetened and unsweetened), beer (200 and 330 cc), non-alcoholic beer (200 and 330 cc), wine, sprits, mixed alcoholic drinks, energy drinks, sports drinks (200 and 330 cc), meal replacement shakes and other beverages. Total fluid intake was considered to be the sum of all types of beverage.

The amount of water in each beverage was estimated using the percentage of water values from the United States Department of Agriculture (USDA) online database [24]. All of the analyses were performed taking into account the mL of water content in each beverage.

### 2.3. Urine Collection

Participants provided a 24-h urine sample, and trained personnel recorded the volume, the day it was provided and the mean environmental temperature of the collection day. Participants were advised that, in the morning, the first urine of the collection day should be discarded, and the first urine sample of the following day included, thus concluding the 24-h cycle. After receiving the urine sample, the trained personnel aliquoted the samples and kept them at −80 °C. Urine osmolality (Uosm) was measured (mOsm/kg) before 31 weeks of freezing using the refractive index method and the osmometer ARKRAY OM6050 (Arkay Global Business, Kyoto, Japan) Osmo Station. Urine osmolality is a measure of the number of dissolved particles per unit of water in urine. Some of these particles can include chloride, glucose, potassium, sodium or urea. In the context of nutrition, the osmolality of a 24-h urine sample reflects the self-regulating activity of renal concentration or dilution mechanisms during a 24-h period. It measures the functional surplus of water and characterizes 24-h hydration status [25].

### 2.4. Assessment of Other Covariates

At baseline and in each visit during the follow-up, questionnaires were administered about lifestyle variables, educational achievement, history of illness, and medication use. Physical activity was assessed using a validated Spanish version of the Minnesota Leisure-Time Physical Activity questionnaire [26]. Trained personnel took the anthropometric measurements. Weight and height were measured with light clothing, and no shoes with calibrated scales and a wall-mounted stadiometer (Certified scale BARYS with stadiometer T2), respectively. Trained dietitians completed a 137-item semi-quantitative and validated [27] FFQ in a face-to-face interview with the participant. Energy and protein intake were estimated using a Spanish food composition table [28,29]. In addition, dietitians administered a 17-item MedDiet screener, adapted from the 14-item questionnaire validated for the PREDIMED study [30], to assess the degree of adherence to the traditional MedDiet.

### 2.5. Statistical Analysis

Beverages and total fluid intake (mL/day) and demographic characteristics are presented as a means (SD) for continuous variables or percentages (numbers) for dichotomous variables. Student’s *t*-test or Pearson’s χ^2^ tests were used to compare the quantitative or categorical general characteristics of the participants. 

To assess relative validity, the total daily fluid intake assessed by the fluid-specific questionnaire was compared to the urine osmolality and the 24-h urine volume values. Associations among these variables were assessed using the correlational analysis Bland–Altman agreement method. A total of 160 participants were included in the validity analysis. A stepwise method was used to select only the significant predictors for urine osmolality. The list of covariates that were not kept in the final model (i.e., did not contribute significantly) to the model urine osmolality were: sex, height, weight, center of recruitment, intervention group, total protein intake, MedDiet adherence, leisure-time physical activity, mean environmental temperature, urine albumin and urine creatinine. The covariates that were kept in the model included age, BMI and total energy consumption. The model for the 24-h urine volume analysis included age and total energy intake. No predictor interactions were found with any of the aforementioned variables. Quintiles of total water intake, osmolality and 24-h urine volume were calculated. The osmolality and 24-h urine volume values were adjusted by the same covariates as were used in the validity analysis. The degree of gross misclassification in the fluid-specific questionnaire with respect to the adjusted osmolality and adjusted 24-h urine volume values was evaluated using contingency tables. The proportions of correctly categorized subjects in the same or adjacent quintiles, and also the individuals classified in extreme quintiles were calculated.

The repeatability of the fluid-specific questionnaire was examined by comparing baseline, and six-month and 12-month values (in 45 and 34 individuals, respectively) with paired Student’s *t*-tests. For a comparison between repeatedly measured variables of consumption of each type of beverage and total fluid intake during time (baseline, six month and one year), a linear mixed-effect model for repeated measures was used. In order to avoid the effect of the intervention on beverage and total-water intake, only individuals from the control group were included in the repeatability analysis. 

The level of significance for all the statistical tests was set at *p* < 0.05 for bilateral contrast. Analyses were performed using JMP version 12.1.0 (SAS Institute Inc., Cary, NC, USA) and with SPSS software, version 22.0 (SPSS Inc., Chicago, IL, USA).

## 3. Results

A total of 160 participants (68 men and 92 women) with a mean age of 65.3 years (range 55 to 75 years) were included in the present analysis. Height and weight, but not BMI, were significantly different between men and women. Such lifestyle variables as leisure-time physical activity, MedDiet adherence and total energy consumed were different between genders. Levels of urine osmolality, urine creatinine and urine albumin were higher in men. Women took significantly more pain relief pills and tranquilizers than men. The general characteristics of the study participants are summarized in Table 1.

### 3.1. Relative Validity of the Questionnaire

Total daily fluid intake from beverages assessed by the specific questionnaire was negatively associated with age and urine osmolality, and positively associated with BMI and total energy intake (*R*^2^: 0.20; *p* < 0.001). The Bland–Altman analysis showed relatively good agreement between total daily fluid intake assessed using the fluid-specific questionnaire, and urine osmolality and 24-h volume with parameter estimates of −0.65 and 0.22, respectively. The validity results for the total daily fluid intake assessed with the specific questionnaire are presented in Table 2. The Bland–Altman plot showing the relationship between total daily fluid intake and 24-h urine volume is shown in a supplementary file (Figure S2).

The percentage of gross misclassification (both over-and underestimation by the fluid-specific questionnaire) as indices of validity of the fluid-specific questionnaire in categorizing individuals was performed (Supplementary Materials Table S1). Osmolality analysis classified 66% of the individuals into the same or the adjacent quintile (±1 quintile) with both methods. A total of 4.4% of the individuals were classified into quintiles at opposite ends of the scale (highest quintile of total water from beverage intake and lowest quintile of osmolality). A total of 6.9% of the population was classified into the lowest quintile of total water intake and the highest quintile of osmolality, suggesting that the total water intake from fluids may have been underestimated. In the 24-h urine volume analysis, 65.7% of the individuals were categorized in the same or the adjacent quintile (± 1 quintile) by both methods. A total of 4.4% and 1.3% of the population studied were misclassified in extreme quintiles (the highest quintile of total water intake and the lowest 24-h urine volume quintile, and the lowest of the total water intake and the highest 24-h urine volume quintiles, respectively).

### 3.2. Repeatability of the Questionnaire

Table 3 shows the repeatability of the fluid-specific questionnaire measurements for each type of beverage analyzed (baseline vs. six months and baseline vs. one year). The consumption in mL/day of each type of beverage and total daily fluid intake at baseline, six months and one year is described. The differences in the consumption (mL/day) between baseline and six months and baseline and one year, and differences in the consumption during all the visits are also shown in the table. No significant differences were found in the fluid consumption from beverages between the baseline and six months or one-year assessments.

## 4. Discussion

The main objective of the present analysis was to assess the relative validity and repeatability of a fluid-specific questionnaire designed to measure the habitual consumption of drinking water and different types of beverage. We report for the first time that the use of a fluid-specific questionnaire in Spanish and designed for the Spanish population seems to be highly repeatable, and relatively valid for estimating the daily intake of water from beverages. This tool may be useful for clinicians and researchers interested in assessing habitual water-drinking and beverage-consumption patterns, particularly in large-scale investigations, in which other resource-intensive dietary intake assessment techniques are not so accurate [31]. 

Although the present fluid-specific questionnaire is the only one to have been validated in Spanish, other questionnaires designed to evaluate beverage intake have been published and validated by a variety of different methods [17,21,31,32]. In 2009, Neuhouser and coworkers developed a questionnaire for assessing the consumption of snacks and beverages, mainly sweetened beverages, by young adolescents [31]. The participants filled in the self-reported beverage questionnaire and also a four-day dietary record. This second method was compared with the beverage questionnaire to assess its validity. The same method was used by Hedrick in 2010 to validate a questionnaire designed to assess the intake of water and caloric beverages [21]. This study used the energy intake from the four-day food record as a method for validating the fluid questionnaire. Although urine samples were collected, they were used to objectively determine total fluid intake and to encourage accurate self-reporting, not for purposes of validation. To date, and to the best of our knowledge, only one questionnaire has been validated using hydration indices with 24-h urine samples [32]. In 2012, Malisova and colleagues developed a “water balance questionnaire”, designed to evaluate water drinking and also water intake from solids and other beverages [32]. For validation purposes, urine was collected from 40 healthy adults and osmolality, 24-h volume, specific gravity, pH and color were evaluated. Although all of these indices have been demonstrated to be biomarkers of hydration, nowadays there is still no biomarker universally accepted as the “gold standard” [33,34]. Nevertheless, in the Malisova study, urine osmolality was proposed as the most promising urine biomarker of all the ones used [32,35]. In the present analysis, the 24-h urine samples were frozen for a few weeks, and the freezing-point depression method could not be used for assessing osmolality. Even though the method used in our study was not the same as the one used in the previously mentioned paper, the validation results were very similar in both studies. The results were also similar for the 24-h urine volume as a biomarker of hydration status. Urine volume in both of our studies and Malisova’s was found to be significantly related to hydration, but not as strongly as to urine osmolality.

In our study, only 1% to 7% of the subjects were misclassified into extreme quintiles. We found that total water intake was considerably underestimated with the fluid-specific questionnaire in comparison with adjusted-osmolality values. This may be because beverages, mainly drinking water, are consumed during the day and often between meals, so they are not perceived as an important food by the participants and tend to be underestimated [18,19].

The second important outcome of the present study is that the repeatability of the Spanish fluid-specific questionnaire was tested. No differences were found for any of the beverages or in the total daily fluid intake at the different times of evaluation (baseline versus six months or one year), either during all the visits as repeated measures. Therefore, beverage intake and patterns can be compared over time. 

The test–retest interval between the three evaluation times of the questionnaire is a factor that has an important influence on repeatability [27]. If the interval is too short, the following evaluations can be influenced by the memory of the first answers, and repeatability will be overestimated. On the other hand, if the interval is too long, the drinking patterns may have changed, which could lead to an underestimation of repeatability [36]. According to a comprehensive review, the time intervals in reports using FFQs range between 2 h and 15 years [37]. In the present analysis, we chose time intervals of six months and one year to prevent the types of bias mentioned above.

This study has several strengths. The ability to accurately assess the validity and repeatability of a questionnaire relies on having a large sample [38] and using multiple statistical methods, which has been achieved in this present study. The second strength is the use of hydration biomarkers instead of dietary intake methods to determine the validity of the analysis. Biomarkers make it possible to improve validation, as they avoid bias caused by measurement errors (memory of the interviewers, errors in estimating food intake), which impact on the statistical power of the study. Another important strength of the present analysis is that the questionnaire was completed by trained dietitians. By avoiding the use of self-reported data we significantly reduced the risk of underreporting errors. However, the study also has several limitations. The present questionnaire may underestimate certain beverage categories because of the serving sizes established (for example, water intake (tap and bottled)). However, estimated mean daily water intake and also total daily fluid intake are very similar to those reported in 2014 in a Spanish population [39], and the present findings did not indicate a ceiling effect. Due to the fact that our population was middle-aged and elderly rather than healthy individuals, future studies should focus on healthy adults and children and other minorities to determine if the fluid-specific questionnaire is a valid tool across other population groups. Another limitation was the lack of a measure to assure the completeness of the 24 h urine samples. However, at the moment the urine was brought in, we asked the participants whether they had followed the instructions and whether they had had any problem with the collection. A final limitation was the use of frozen samples. It has been suggested that freezing urine samples generates urinary sediments that consist predominantly of endogenous calcium oxalate dehydrate and amorphous calcium crystals [40] and that this may account for the changes in osmolality observed after freezing. However, several studies have shown that the changes in frozen urine osmolality are trivial and physiologically irrelevant, especially because daily variations in urine osmolality are considerably larger than these changes [41,42]. The long-term stability and measurement validity for frozen urine were found to be good without the addition of a preservative. The prospective storage of frozen urine aliquots, even exceeding 10 years, appears to be an acceptable and valid tool in epidemiological settings for subsequent urine analysis [43]. Nevertheless, in the present study, we measured osmolality levels in a subsample of urine just after the collection (*n* = 59), without freezing, and no significant differences were found (data not shown).

## 5. Conclusions

The present fluid-specific questionnaire appears to be a relatively valid and a highly reliable tool for assessing intake of water and other types of beverages in Spanish adults. The Spanish beverage intake assessment questionnaire may help nutrition researchers and clinicians to evaluate beverages, patterns and changes in consumption and their influence on health or disease.

## Figures and Tables

**Table 1:** General characteristics of the study population.

Variables	All Population (*n* = 160)	Men (*n* = 68)	Women (*n* = 92)	*p*-Value ^a^
Age, years	65.3 (4.9)	64.5 (5.9)	65.9 (3.9)	0.097
Height, m	1.62 (0.09)	1.69 (0.06)	1.56 (0.06)	<0.001
Weight, kg	86.7 (14.3)	94.3 (12.5)	81.9 (12.9)	<0.001
BMI, kg/m^2^	33.0 (4.3)	32.9 (3.6)	33.1 (4.7)	0.328
Leisure-time physical activity, METs/week	3123 (2804)	4006 (2945)	2471 (2518)	<0.001
Mediterranean diet score, (0–17 points)	9.2 (2.5)	8.5 (2.6)	9.8 (2.3)	<0.005
Total energy intake, kcal/day	2229 (551)	2330 (606)	2155 (497)	<0.005
Total protein intake, g/day	134 (357)	189 (545)	93 (22)	0.276
Urine volume, mL/day	1722 (651)	1762 (698)	1693 (616)	0.506
Urine osmolality, mOsm/kg	551 (211)	631 (204)	492 (196)	<0.001
Urine albumin, mg/dL	13.8 (31.8)	20.0 (39.5)	9.0 (23.4)	0.047
Urine creatinine, μmol/dL	7718 (3760)	9440 (4204)	6431 (2783)	<0.001
Urine albumin to creatinine ratio, mg/g	17.1 (43.4)	22.2 (53.2)	13.2 (33.9)	0.228
Use of medications, % (*n*)				
Aspirin	24.4 (39)	26.5 (18)	22.8 (21)	0.596
Pain relief	33.7 (54)	17.6 (12)	45.6 (42)	<0.005
Tranquilizers	27.5 (44)	17.6 (12)	34.8 (32)	0.016
Vitamin/minerals	6.9 (11)	2.9 (2)	9.8 (9)	0.091
Heart problems	4.4 (7)	5.9 (4)	3.3 (3)	0.423
Antihypertensive agents	79.4 (127)	82.3 (56)	77.2 (71)	0.423
Statins	56.9 (91)	50.0 (34)	62.0 (57)	0.131
Insulin	6.2 (10)	5.9 (4)	6.5 (6)	0.869
Oral anti-diabetic drugs	30.0 (48)	30.9 (21)	29.3 (27)	0.834
Others	68.1 (109)	63.2 (43)	71.7 (66)	0.254

Data expressed as means (SD) or percentages (*n*). Abbreviations: BMI, body mass index. ^a^ *p*-Values for comparisons between groups were tested by Student’s *t*-test or χ^2^ as appropriate.

**Table 2:** Parameter estimates for two candidate models (osmolality and urine volume) with similar predictive ability of total daily beverage intake.

Term	Parameter Estimate	Standardized β *	Standard Error	*p*-Value	*R*^2^
Intercept	2278				
Osmolality	−0.65	−0.26	0.18	0.0005	0.20
Age	−25.13	−0.23	7.94	0.0019	
BMI	23.86	0.15	11.38	0.0376	
Total energy	0.27	0.25	0.08	0.0007	
Intercept	2455				
Urine volume	0.22	0.27	0.06	0.0003	0.20
Age	−26.03	−0.24	7.93	0.0013	
Total energy	0.24	0.23	0.07	0.0019	

* Standardized beta weights are indicative of effect size.

**Table 3:** Repeatability of the beverage intake assessment questionnaire.

Beverage Category	Baseline (mL/day) (*n* = 67)	Baseline vs. 6 Months			Baseline vs. 1 Year			
		Mean (SD)			Mean (SD)			
		6 Months (mL/day) (*n* = 45)	Differences from Baseline	*p*-Value ^a^	1 Year (mL/day) (*n* = 34)	Differences from Baseline	*p*-Value ^a^	*p-*Value ^b^
Tap water	289 (571)	449 (657)	62 (413)	0.32	360 (577)	−23 (502)	0.79	0.389
Bottled water	755 (539)	773 (714)	80 (505)	0.29	813 (612)	125 (577)	0.22	0.905
Natural fruit juices	39 (69)	27 (54)	1 (79)	0.92	28 (61)	16 (75)	0.21	0.537
Bottled fruit juices	26 (93)	22 (52)	7 (46)	0.33	19 (44)	7 (29)	0.16	0.880
Natural vegetable juices	6 (28)	16 (36)	9 (29)	0.05	1 (9)	−1 (16)	0.79	0.073
Bottled vegetable juices	14 (69)	4 (14)	−5 (32)	0.25	4 (18)	−7 (41)	0.35	0.451
Whole milk	24 (92)	3 (17)	−16 (89)	0.22	25 (74)	4 (125)	0.86	0.284
Semi-skimmed milk	43 (81)	67 (126)	8 (125)	0.67	63 (123)	9 (131)	0.69	0.467
Skimmed milk	95 (146)	59 (107)	−19 (145)	0.38	54 (102)	−36 (151)	0.17	0.192
Drinking yogurt (100 cc)	13 (32)	10 (32)	−6 (32)	0.23	8 (23)	−6 (25)	0.18	0.765
Drinking yogurt (200 cc)	6 (31)	13 (46)	5 (61)	0.58	10 (42)	4 (54)	0.56	0.562
Milkshakes	0 (2)	0 (0)	0 (3)	0.32	2 (10)	1 (11)	0.53	0.289
Vegetable drinks	21 (80)	9 (39)	5 (49)	0.47	38 (128)	33 (133)	0.16	0.330
Soups	36 (30)	34 (35)	0 (45)	0.93	50 (56)	17 (52)	0.07	0.144
Jellies and sorbets	2 (11)	1 (5)	−1 (9)	0.61	1 (4)	−1 (7)	0.64	0.594
SSBs (200 cc)	11 (34)	11 (38)	3 (31)	0.53	7 (32)	−3 (18)	0.32	0.862
SSBs (330 cc)	8 (39)	18 (90)	6 (92)	0.68	10 (51)	5 (54)	0.59	0.707
ASBs (200 cc)	7 (48)	21 (118)	11 (134)	0.57	39 (158)	37 (157)	0.18	0.363
ASBs (330 cc)	42 (164)	8 (32)	−54 (200)	0.08	18 (45)	−61 (215)	0.11	0.292
Espresso sweetened	16 (31)	9 (20)	−4 (31)	0.33	9 (18)	−4 (20)	0.25	0.287
Espresso unsweetened	24 (35)	36 (38)	7 (36)	0.22	31 (37)	2 (23)	0.62	0.223
White coffee sweetened	23 (63)	5 (25)	−3 (40)	0.66	4 (21)	−2 (36)	0.69	0.063
White coffee unsweetened	9 (31)	3 (18)	−5 (35)	0.32	7 (28)	3 (36)	0.57	0.492
Tea sweetened	7 (29)	2 (13)	−7 (26)	0.09	14 (48)	2 (65)	0.82	0.246
Tea unsweetened	25 (87)	34 (105)	17 (79)	0.15	19 (51)	−2 (54)	0.82	0.718
Other infusions sweetened	27 (96)	13 (66)	12 (67)	0.23	17 (55)	15 (56)	0.13	0.646
Other infusions unsweetened	34 (91)	51 (107)	14 (90)	0.30	54 (139)	12 (106)	0.51	0.613
Beer (200 cc)	18 (67)	10 (32)	−12 (68)	0.24	1 (4)	−18 (71)	0.13	0.266
Beer (330 cc)	32 (91)	47 (119)	2 (131)	0.92	26 (66)	6 (69)	0.63	0.580
Non-alcoholic beer (200 cc)	5 (25)	11 (39)	−8 (45)	0.21	5 (24)	−4 (20)	0.28	0.581
Non-alcoholic beer (330 cc)	13 (54)	3 (14)	4 (22)	0.25	2 (15)	−11 (58)	0.25	0.283
Wine	35 (63)	41 (68)	−7 (60)	0.46	60 (85)	9 (55)	0.35	0.257
High alcoholic content beverages	1 (3)	1 (4)	0 (3)	0.66	1 (2)	0 (3)	0.26	0.988
Mixed alcoholic beverages	1 (6)	0 (3)	0 (0)	-	0 (0)	0 (3)	0.32	0.388
Energy drinks	0 (0)	0 (0)	0 (0)	-	0 (0)	0 (0)	-	-
Sports drinks (200 cc)	0 (3)	0 (0)	0 (4)	0.32	0 (0)	0 (0)	-	0.558
Sports drinks (330 cc)	0 (0)	2 (13)	2 (13)	0.32	0 (0)	0 (0)	-	0.328
Meal replacement shakes	0 (0)	0 (0)	0 (0)	-	0 (0)	0 (0)	-	-
Other drinks	0 (0)	0 (0)	0 (0)	-	0 (0)	0 (0)	-	-
Total water intake	1711 (64)	1816 (498)	106 (475)	0.14	1804 (435)	128 (559)	0.19	0.477

Data expressed as means (SD). ^a^ *p*-values for comparisons between groups were tested by Student’s *t*-test; ^b^ *p*-Values for comparisons between repeated measures were tested by linear mixed models test.

## Supplementary Materials

The following are available online at http://www.mdpi.com/2072-6643/8/8/475/s1, Figure S1: The beverage intake assessment questionnaire in Spanish (validated tool); Figure S2: Bland–Altman plots showing the relationship between total daily water intake (mL/day) and 24-h urine volume (mL/day); Table S1: Contingency tables for the gross misclassification between quintiles of total daily water intake and (A) osmolality adjusted or (B) 24-h urine volume adjusted.

## Abbreviations

SSBs, sugar-sweetened beverages; ASBs, artificially-sweetened beverages; FFQ, food frequency questionnaire; CVD, cardiovascular diseases; MetS, metabolic syndrome; MedDiet, Mediterranean diet; USDA, United States Department of Agriculture; SD, Standard Deviation; ANOVA, analysis of variance; BMI, body mass index.
