# Supplementary material for: Beverage Intake Assessment Questionnaire: Relative Validity and Repeatability in a Spanish Population with Metabolic Syndrome from the PREDIMED-PLUS Study
Source: Nutrients. 2016 Jul 30;8(8):475. doi: 10.3390/nu8080475 (PMC4997388; doi:10.3390/nu8080475)
Supplement: Supplementary file 1 [file nutrients-08-00475-s001.docx]

Supplementary Materials: Beverage Intake Assessment Questionnaire: Relative Validity and Repeatability in a Spanish Population with Metabolic Syndrome from the
PREDIMED-PLUS Study

Cíntia Ferreira-Pêgo, Mariela Nissensohn, Stavros A. Kavouras, Nancy Babio,
Lluís Serra-Majem, Adys Martín Águila, Andy Mauromoustakos, Jacqueline Álvarez Pérez
and Jordi Salas-Salvadó


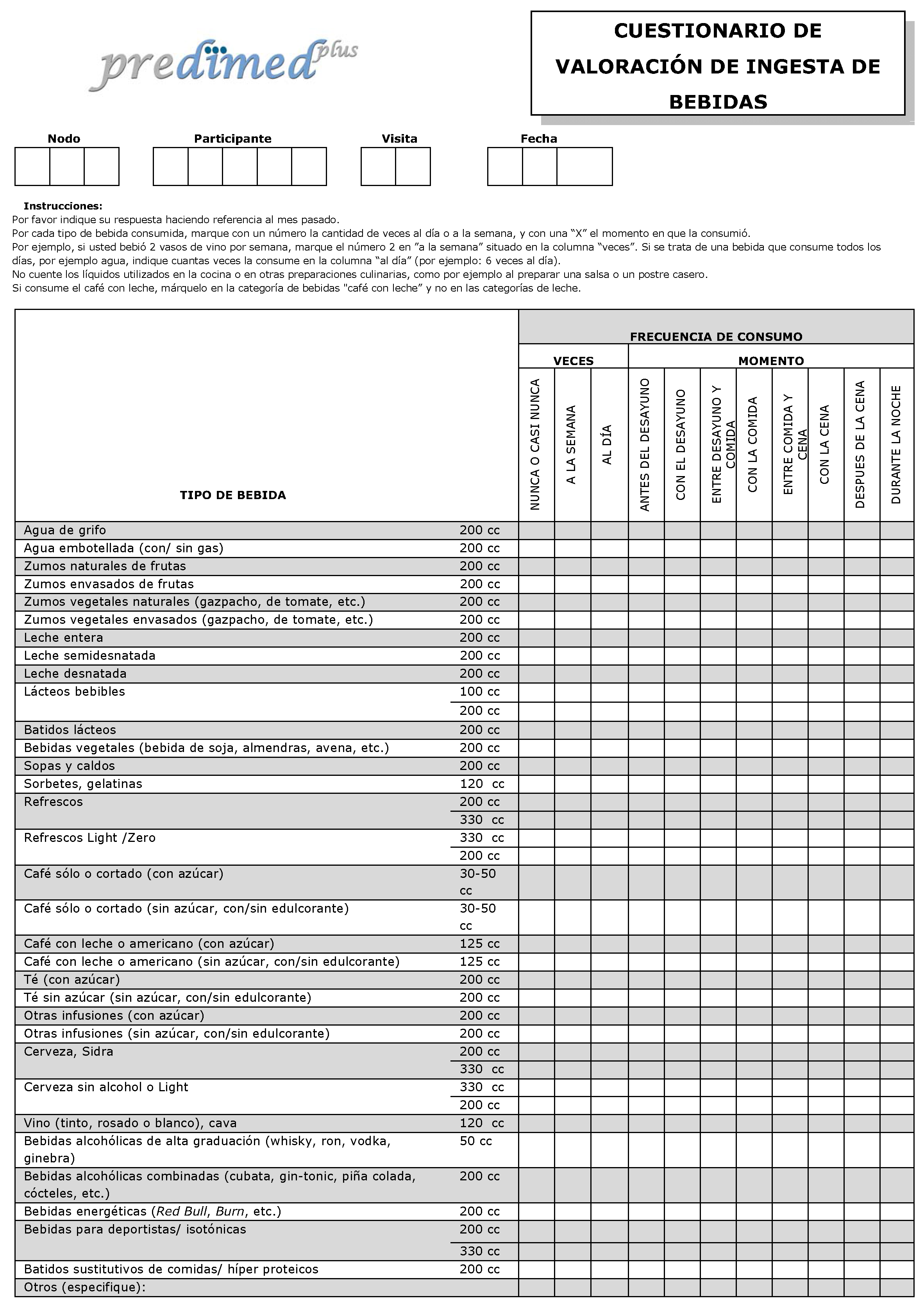


**Figure S1.** The beverage intake assessment questionnaire in Spanish (validated tool).


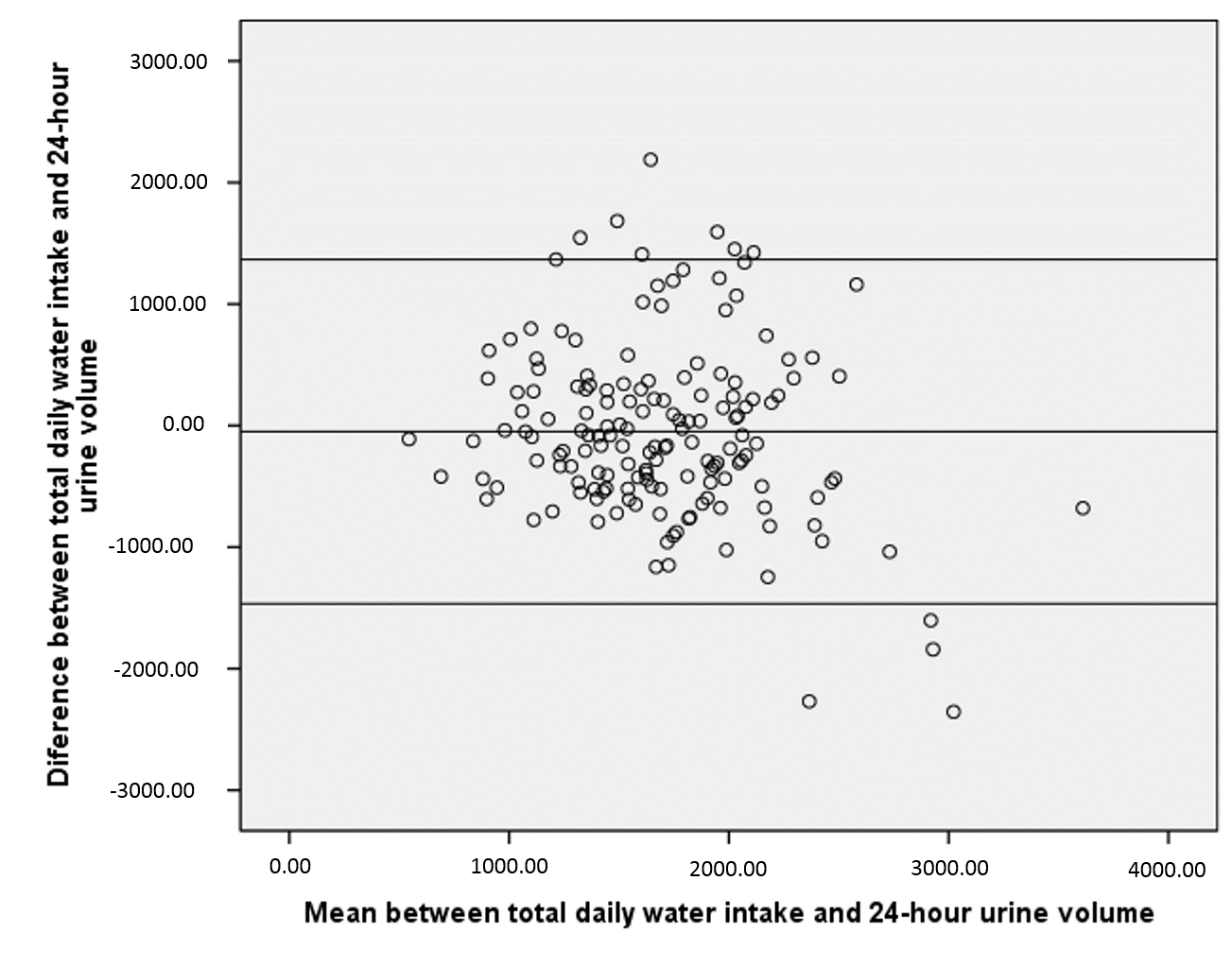


**Figure S2.** Bland-Altman plots showing the relationship between total daily water intake (mL/day) and 24-h urine volume (mL/day).

**Table S1.** Contingency tables for the gross misclassification between quintiles of total daily water intake and (**A**) osmolality adjusted or (**B**) 24-h urine volume adjusted.

| Quintiles of total daily water intake (%) | (**A**) **Quintiles of Osmolality Adjusted (%)** | | | | | |
| --- | --- | --- | --- | --- | --- | --- |
|  |  | **Q1** | **Q2** | **Q3** | **Q4** | **Q5** |
|  | **Q1** | 6.4 | 1.9 | 2.4 | 2.4 | 6.9 |
|  | **Q2** | 3.1 | 7.7 | 4.4 | 2.4 | 2.4 |
|  | **Q3** | 2.4 | 6.3 | 5.8 | 3.8 | 1.8 |
|  | **Q4** | 3.6 | 1.8 | 6.3 | 6.9 | 1.3 |
|  | **Q5** | 2.4 | 2.4 | 3.1 | 4.4 | 7.7 |
|  | (**B**) **Quintiles of 24-h Volume Adjusted (%)** | | | | | |
|  |  | **Q1** | **Q2** | **Q3** | **Q4** | **Q5** |
|  | **Q1** | 8.1 | 4.4 | 5.0 | 1.3 | 1.3 |
|  | **Q2** | 3.1 | 5.0 | 5.0 | 3.1 | 3.8 |
|  | **Q3** | 2.5 | 5.0 | 3.8 | 5.0 | 3.8 |
|  | **Q4** | 1.9 | 1.3 | 4.4 | 5.0 | 7.5 |
|  | **Q5** | 4.4 | 4.4 | 1.9 | 5.6 | 3.8 |
